# Magnetic resonance imaging datasets with anatomical fiducials for quality control and registration

**DOI:** 10.1038/s41597-023-02330-9

**Published:** 2023-07-12

**Authors:** Alaa Taha, Greydon Gilmore, Mohamad Abbass, Jason Kai, Tristan Kuehn, John Demarco, Geetika Gupta, Chris Zajner, Daniel Cao, Ryan Chevalier, Abrar Ahmed, Ali Hadi, Bradley G. Karat, Olivia W. Stanley, Patrick J. Park, Kayla M. Ferko, Dimuthu Hemachandra, Reid Vassallo, Magdalena Jach, Arun Thurairajah, Sandy Wong, Mauricio C. Tenorio, Feyi Ogunsanya, Ali R. Khan, Jonathan C. Lau

**Affiliations:** 1 Imaging Research Laboratories, Robarts Research Institute, Western University, London, Canada; 2 School of Biomedical Engineering, Western University, London, Canada; 3 Department of Clinical Neurological Sciences, Division of Neurosurgery, Western University, London, Canada; 4 Graduate Program in Neuroscience, Schulich School of Medicine and Dentistry, Western University, London, Canada; 5 Department of Medical Biophysics, Schulich School of Medicine and Dentistry, Western University, London, Canada; 6 Department of Anatomy and Cell Biology, Schulich School of Medicine and Dentistry, Western University, London, Canada; 7 Michael G. DeGroote School of Medicine, McMaster University, Hamilton, Canada; 8 School of Biomedical Engineering, Faculty of Applied Science and Faculty of Medicine, The University of British Columbia, Vancouver, Canada; 9 Centre for Functional and Metabolic Mapping, Robarts Research Institute, Western University, London, Canada

**Keywords:** Brain, Databases, Parkinson's disease

## Abstract

Tools available for reproducible, quantitative assessment of brain correspondence have been limited. We previously validated the anatomical fiducial (AFID) placement protocol for point-based assessment of image registration with millimetric (mm) accuracy. In this data descriptor, we release curated AFID placements for some of the most commonly used structural magnetic resonance imaging datasets and templates. The release of our accurate placements allows for rapid quality control of image registration, teaching neuroanatomy, and clinical applications such as disease diagnosis and surgical targeting. We release placements on individual subjects from four datasets (N = 132 subjects for a total of 15,232 fiducials) and 14 brain templates (4,288 fiducials), totalling more than 300 human rater hours of annotation. We also validate human rater accuracy of released placements to be within 1 – 2 mm (using more than 45,000 Euclidean distances), consistent with prior studies. Our data is compliant with the Brain Imaging Data Structure allowing for facile incorporation into neuroimaging analysis pipelines.

## Background & Summary

Open resources available for reproducible, quantitative assessment of brain correspondence have been limited^1^. The most common metrics employed for the purpose of examining the quality of image registration, including the Jaccard similarity and Dice kappa coefficients, compute the voxel overlap between regions of interest (ROIs), which have been shown to be insufficiently sensitive when used in isolation or in combination for validating image registration strategies^1^. The ROIs used in voxel overlap are often larger subcortical structures that are readily visible on magnetic resonance imaging (MRI) scans (e.g., the thalamus, globus pallidus, and striatum), and thus lack the ability to detect subtle misregistration between images which may be crucial where millimetric differences in variability should be accounted for^1–5^.

Inspired by classic stereotactic methods, our group created, curated, and validated a protocol for the placement of anatomical fiducials (AFIDs) on structural MRI scans of the human brain^2^. The protocol involves the placement of 32 AFIDs found to have salient features that allow for accurate localization. The AFIDs are described using three-dimensional (x, y, and z) Cartesian coordinates and thus correspondence between points can be computed using Euclidean distances across a variety of applications. After a brief tutorial, AFIDs have been shown to be highly reproducible even when performed by individuals with no prior knowledge of medical images, neuroanatomy, or neuroimaging software. This was shown in separate studies where placements were performed on publicly available templates and datasets^2^ and a clinical neuroimaging dataset^3^.

The AFID protocol provides a metric that is independent of the registration itself while offering sensitivity to registration errors at the scale of millimeters (mm). This margin is crucial in neuroimaging applications (including morphometric analysis and surgical neuromodulation), where a few mm may represent the difference between optimal and suboptimal therapy.

**Fig. 1:**
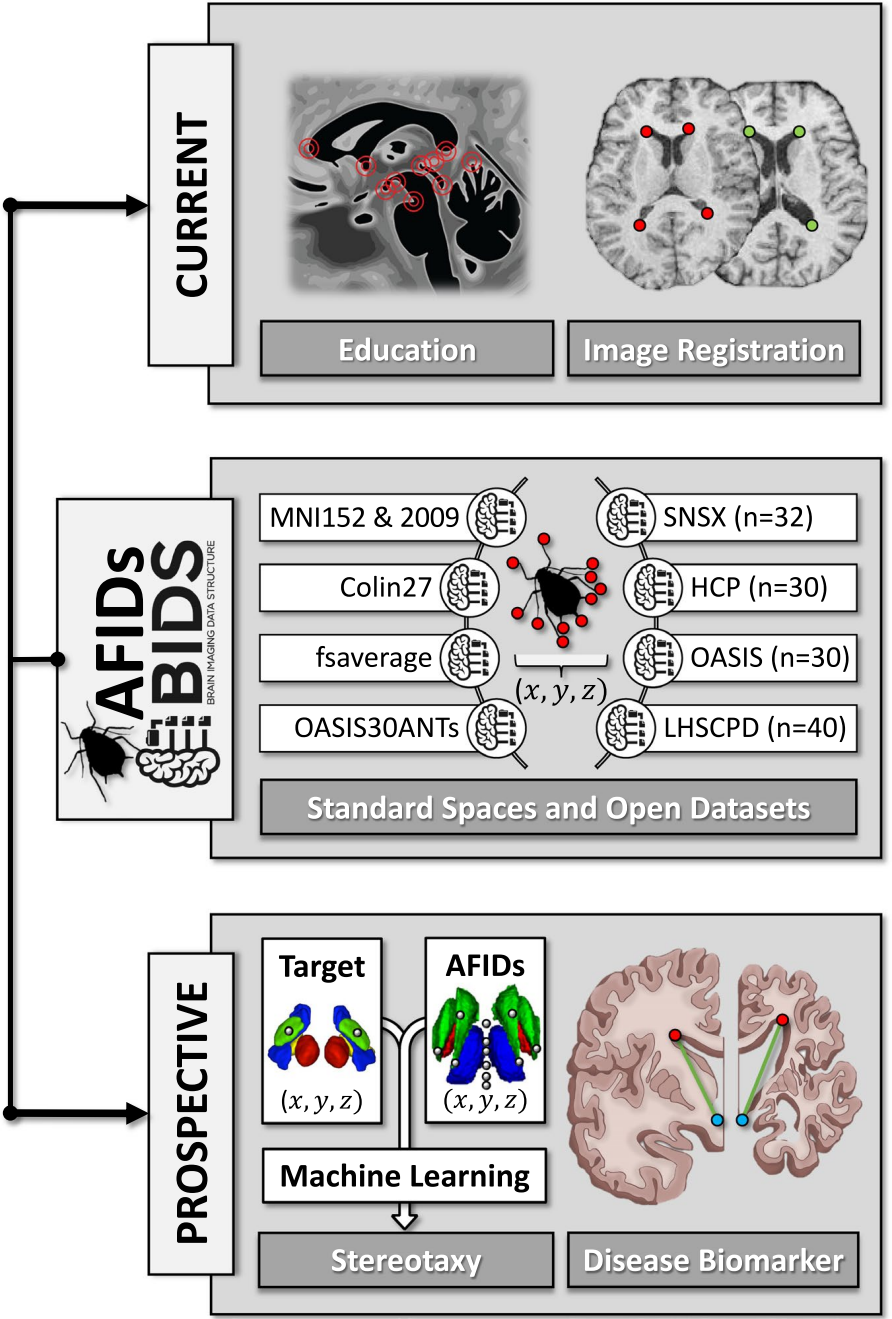
Current and prospective applications of curated anatomical fiducial (AFID) placements. Top Panel: Current applications in neuroanatomy education and image registration. Middle Panel: Released healthy and pathologic datasets and templates (detailed descriptions can be found in text). Bottom Panel: Prospective applications of AFIDs in stereotactic targeting and as a disease biomarker.

The aim of this data descriptor is to provide the community with curated AFID placements and their associated MRI images. We release annotations on four datasets (N = 132; 15,232 fiducials) including healthy subjects and patients with neurological disorders, and 14 commonly used MRI templates (4,288 fiducials), totalling more than 300 human rater hours of manual annotation of neuroanatomical structures. Descriptions of the datasets and templates are provided in subsequent sections. We highlight current and prospective applications of our released data in Fig. 1.

### Current applications

#### Registration assessment

We share our curated AFID annotations for a wide variety of datasets and templates of varying field strengths. This diversity of datasets will facilitate the testing and validation of image registration algorithms that can be used in many contexts. The user can select the datasets and templates that are in line with their neuroimaging application, then use the curated annotations to assess image registration quantitatively. For instance, AFIDs have been used to evaluate the process of iterative deformable template creation^6,7^, showing that error metrics generated from AFIDs converged differently as a function of template iterations and registration method (i.e., linear vs non-linear). Sharing the AFID placements and their associated images in the Brain Imaging Data Structure (BIDS) format aids in the convenience we strive to provide for the end-user and neuroimaging application developer^2,3,6,7^.

#### Education

**Fig. 2:**
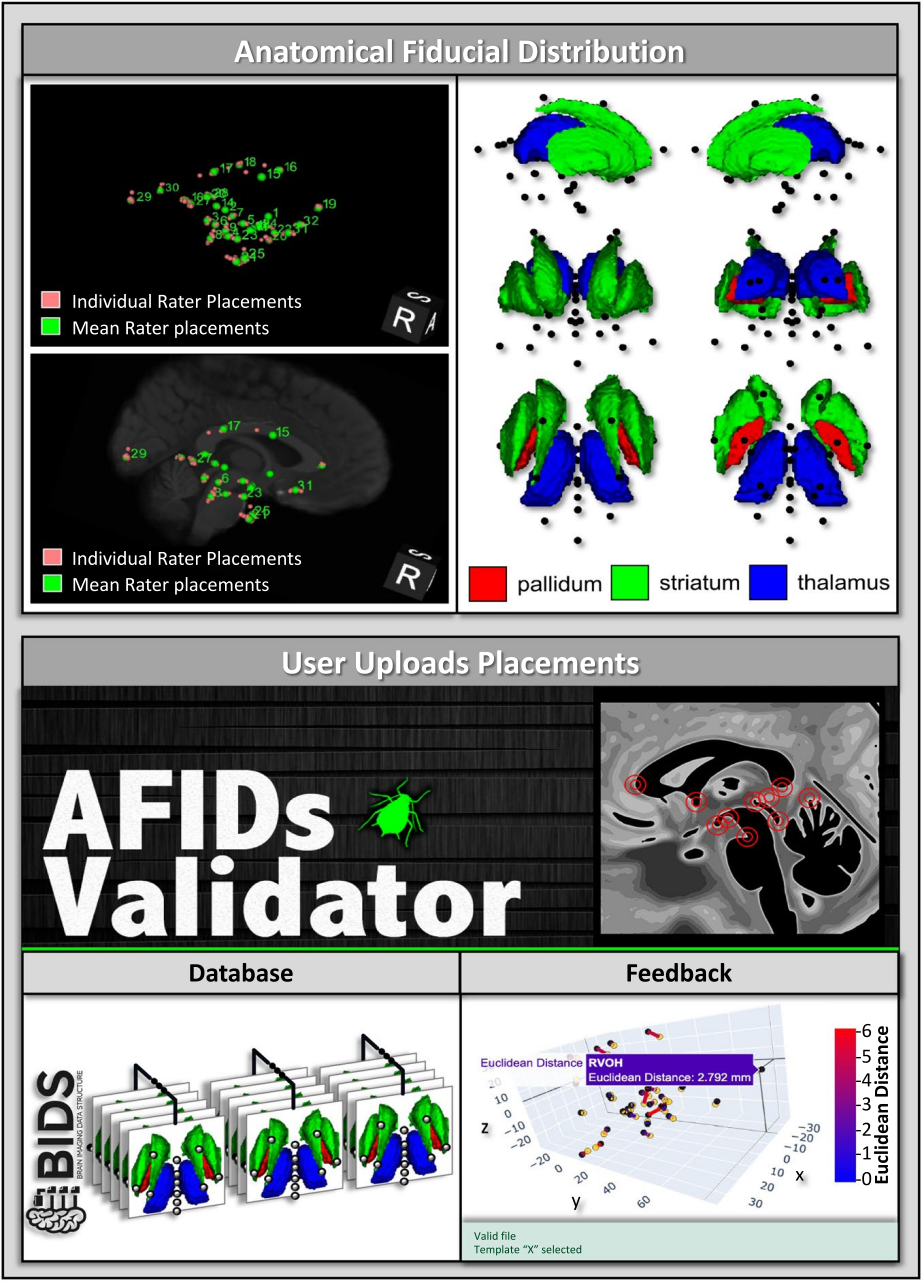
Curated AFID locations within the brain and usage of the AFIDs validator website. Top Panel: Distribution of AFIDs overlayed on one of the released templates. We also show major subcortical structures with AFIDs (black points) in various anatomical views. Bottom Panel: Uses and outputs from the AFIDs validator website (https://validator.afids.io)^8^. The user decides whether to upload their placements to our database and will receive summary metrics regarding their placements in an interactive 3D coordinate system and tabular format (not shown).

New raters can learn to view and localize anatomical regions using our AFID framework then autonomously compare their placements to the curated normative distribution placements we release here. Our placements have been compiled over the years and can help raters assess accuracy for specific fiducials and subject/template data. To improve user accessibility and navigation of our released AFID annotations and framework, we also release the AFIDs validator (https://validator.afids.io; see Fig. 2)^8^. This tool provides: (1) detailed descriptive and visual documentation of the AFID placement protocol, (2) an interactive way for users to upload placements to a regulated database, and (3) interactive methods to view uploaded placements relative to curated placements almost instantaneously, which helps guide users to improve neuroanatomical understanding and placement accuracy^2,3^.

#### Brain structure and volumetric analyses

AFIDs (and associated images) in our pathological dataset relative to the control can allow for insight into brain morphology and putative biomarkers of neurodegenerative diseases^3^.

### Prospective applications

#### Registration optimization and quality control

The released imaging and AFID placement data may be useful in a few ways for improving neuroimaging pipelines: (1) providing centralized and quality controlled neuroimaging data (from 4 different neuroimaging datasets) allowing for a more accurate and generalizable head-to-head comparison among existing software for image registration, and (2) establishing a new registration metric that can be incorporated into neuroimaging software development workflows to optimize registration algorithm performance and ensure quality control.

#### Automatic and accurate landmark placement

Our curated AFIDs can serve as ground truth placements when training machine learning algorithms to automate brain landmark localization. Among the 32 AFIDs we release are the anterior and posterior commissures (AC and PC, respectively). Downstream applications of automatic localization include automatically computing AC-PC transformation (a common process in neuroimaging studies) and aspects of neurosurgical planning that involve the placement of these anatomical landmarks. The diversity of the released data (both hardware and disease status) will be crucial to the generalizability of such tools.

#### Surgical targeting

We release locally curated ultra-high field (7-Tesla; 7-T) MRI data where small structures like the subthalamic nucleus (STN)^9^ and zona incerta within the posterior subthalamic area are clearly visible^7^. Ground truth locations of surgical targets (x, y, and z) can be related to AFID locations via predictive models. This approach mitigates the lack of access to best-case neuroimaging in clinical settings due to limited access to high-field MRI or motion degradation.

#### Brain anatomy abstraction and anonymization

AFIDs and the distances between them represent an abstraction of brain anatomy in an anonymized way while still allowing for the accurate pooling of data. Other significant anatomical landmarks (representing lesions, tumors, or other structures) can be described in reference to the AFID “coordinate system” we establish using these curated placements.

## Methods

### Rationale for fiducial selection and placement assessments

The current version of the AFID protocol involves the placement of 32 landmarks. They were selected to be easily identified on structural MRI scans across varying field strengths (1.5-T, 3-T, 7-T) and were validated in previous studies^2,3^. During the selection process, regions that were prone to geometric inhomogeneity and distortion were avoided to enhance the accuracy of fiducial placement^2^. There are 10 fiducials that fall on the midline and 11 located laterally on both hemispheres (see Table 1). The AFID protocol includes landmarks representing salient neuroanatomical features mostly located in the subcortex (see Fig. 2).

**Table 1:** Description of curated anatomical fiducials (AFIDs) and related metadata.

Number	Anatomical Fiducial	Acronym	Side
1	anterior commissure	AC	Midline
2	posterior commissure	PC	Midline
3	infracollicular sulcus	ICS	Midline
4	pontomesencephalic junction	PMJ	Midline
5	superior interpeduncular fossa	SIPF	Midline
6,7	superior lateral mesencephalic sulcus	R/L SLMS	Lateral
8,9	inferior lateral mesencephalic sulcus	R/L ILMS	Lateral
10	culmen	CUL	Midline
11	intermammillary sulcus	IMS	Midline
12,13	mammillary body	R/L MB	Lateral
14	pineal gland	PG	Midline
15,16	lateral aspect of frontal horn at AC	R/L LVAC	Lateral
17,18	lateral aspect of frontal horn at PC	R/L LVPC	Lateral
19	genu of corpus callosum	GENU	Midline
20	splenium of the corpus callosum	SPLE	Midline
21,22	anterolateral temporal horn	R/L ALTH	Lateral
23,24	superior anteromedial temporal horn	R/L SAMTH	Lateral
25,26	inferior anteromedial temporal horn	R/L IAMTH	Lateral
27,28	indusium griseum origin	R/L IGO	Lateral
29,30	ventral occipital horn	R/L VOH	Lateral
31,32	olfactory sulcal fundus	R/L OSF	Lateral

*Fiducial localization error (FLE)* is a term described by Fitzpatrick and colleagues^10^ that represents the distance between a fiducial position from its intended location. This term is used when operating image-guidance systems during surgical procedures. In the context of the AFID protocol, and inspired by this extant terminology, we have defined the term anatomical fiducial localization error (AFLE). This value, in mm, can be thought of as the error arising from the placement (i.e., localization) of each fiducial. When used to communicate the accuracy of all AFIDs together, we term it global AFLE. There are three contexts for applying AFLEs: (**1**) **mean AFLE:** rater localization error relative to the intended location defined as the mean placement of all raters for a specific fiducial (termed ground truth AFID in subsequent sections). (**2**) **inter-rater AFLE:** rater localization error calculated as the pairwise distances between different rater placements. If a single rater performed the AFID protocol more than once, then their mean placement coordinates were used for the pairwise distance calculations. (**3**) **intra-rater AFLE:** rater localization error evaluating the precision of multiple placements by a single rater computed as the average pairwise distance between the same rater’s placements.

We also adopt the term *fiducial registration error (FRE)* in the context of the AFID protocol and term it the anatomical fiducial registration error (AFRE). It is important to note that AFRE in our context diverges from the original usage by Fitzpatrick and colleagues^10^ which was restricted to describe registration error at fiducials used to drive image registration (i.e., during landmark-based registration). Computed in mm, AFRE is defined as errors arising from the registration protocol performed between two images (often, but not limited to, subject and template). AFRE is the distance after co-registration between each of the 32 AFIDs placed on a moving image and their counterparts placed on the fixed image (i.e., homologous points). The average AFRE of all fiducials is termed the global AFRE. We also establish nomenclature to differentiate various use cases for AFRE. If an individual rater placement is chosen for subsequent analysis, then we term the resulting AFRE to be the **real-world AFRE** as it is more representative of what would happen in a clinical setting where one rater would apply the AFID protocol. If a ground truth AFID placement is used, then the resulting error is termed **consensus AFRE** as it represents the average placement among a group of raters prior to the image registration step. In this data descriptor, our focus is on releasing the curated AFID placements and not an assessment of registration, so no AFRE metrics are produced. We still felt it would be useful to introduce AFRE as its computation constitutes one of the main applications of AFIDs and our shared datasets for quality control (i.e., in the context of image registration).

### Hardware and software used to curate data

All manually curated AFIDs were placed using the Markups Module of 3DSlicer (an open-source imaging software)^11^. The datasets were curated at different times so a reference to the exact version of 3DSlicer and associated modules will be made under each dataset. 3DSlicer was chosen because it offers a variety of modules, particularly markups and registration modules used for fiducial placement and AC-PC transformation. 3DSlicer stores points placed within its 3D coordinate system overlaid on the image giving the possibility of more accurate localization without the need to interpolate to the nearest voxel. The AFID placements released here for templates and datasets were performed on structural T1w MRI images.

### Performing the AFID protocol

All raters underwent extensive training before being involved in any AFID related studies^2,3^. More specifically, they (1) attended a synchronous session about 3DSlicer and placed all the AFIDs under the supervision of expert raters, (2) were asked to refer to resources found on our AFID protocol website (https://afids.github.io/afids-protocol)^12^ to supplement their learning asynchronously, and (3) uploaded their annotations to the AFIDs validator tool for feedback with further review with an expert to ensure that their annotations were of sufficient quality. We collected demographic data (neuroanatomy, imaging, and 3DSlicer exposure) for raters involved in data curation (see Data Records).

For manual rater placements, the AFID protocol generally began with the placement of the anterior commissure (AC) and posterior commissure (PC) points (AFID01 and 02, respectively), which are defined to be at the center of each commissure. This was then followed by the identification of one or two more midline points (often the pontomesencephalic junction, AFID04, and the genu of corpus callosum, AFID19, are used). After that, an AC-PC transformation is performed, and the rest of the anatomical fiducials are placed. Rater placements deviating from a ground truth fiducial by greater than 10 mm were removed and considered outliers, as these errors are likely to be due to mislabelling and not reflective of true localization accuracy. In addition to subsequent sections, Table 2 provides brief descriptions of the released datasets and templates, information about raters, and AFID placements.

**Table 2:** Summary of templates and datasets released, raters, and anatomical fiducial (AFID) protocol performances.

Template or Dataset	Brief Description	Field Strength (T)	Raters (N)	Total number of AFID annotations	References	
					Imaging	AFID Annotations
MNI2009bAsym	A population group template consisting of 152 individuals commonly used in the neuroimaging literature	1.5	8 novices	8 × 4 (1,024 individual points)	Fonov *et al*.^16^	Lau *et al*.^17^
MNIColin27	A template of a single healthy control subject (N = 1) scanned 27 times and averaged together	1.5			Holmes *et al*.^18^	
Agile12v2016	An ultra-high field template consisting of 12 healthy control averaged subjects created at the Centre for Functional and Metabolic Mapping at Western University	7			Lau *et al*.^17^	
BigBrainSym	Ultra-high resolution histological 3D model of the brain (BigBrain) registered to MNI2009bSym space	N/A; histological	2 experts	2 × 1 (64 individual points)	Amunts *et al*.^19^ Xiao *et al*.^6^	
MNI2009bSym	The symmetric version of the MNI2009bAsym template	1.5			Fonov *et al*.^16^	
PD-25	A multi-contrast MNI template of a Parkinson’s disease (PD) cohort	3			Xiao *et al*.^20^	N/A
TemplateFlow	A centralized resource of open-access templates for neuroimaging studies (tpl-MNI152 -Lin, NLin2009cAsym, NLin2009cSym, NLin6Asym, NLin6Sym, tpl-MNI305, tpl-OASIS30ANTs, tpl-fsaverage)	3+	4 total: 1 expert and 3 novices	4 × 1 (128 individual points)	Ciric *et al*.^22^	N/A
AFIDs-HCP30	A subset of N = 30 healthy control subject images from the Human Connectome Project (HCP) dataset	3	5 experts	3 × 30 (2,880 individual points)	Van Essen *et al*.^13^	N/A
AFIDs-OASIS30	A subset of N = 30 cognitively intact control subject images from the OASIS-1 database selected to exhibit a wide range of normal anatomical variability	3	9 total: 1 expert and 8 novices		Marcus *et al*.^14^	Lau *et al*.^2^
LHSCPD	A set of N = 40 PD patient images acquired at University Hospital (Western University, Canada)	1.5	5 total: 2 expert and 3 novices	5 × 40 (6,400 individual points)	Abbass *et al*.^3^	
SNSX	A set of N = 32 control subject images acquired at the Centre for Functional and Metabolic Mapping atWestern University	7	9 total: 3 expert and 6 novices	3 × 32 (3,072 individual points)	Lau *et al*.^7^	

### AFIDs-HCP30 dataset

#### Subject demographics and imaging protocol

This subset consists of 30 unrelated healthy subjects (age: 21 – 52 years; 15 female) chosen from the Human Connectome Project dataset (HCP). All scans were T1-weighted MR volumes with 1 mm voxels acquired on a 3-T scanner^13^.

#### Rater demographics and AFID placements

The AFID protocol was performed a total of three times on this dataset (2,880 fiducials). Five expert raters were involved with annotations (3DSlicer 4.10.0). Each scan within this dataset was assigned for annotation by three expert raters.

### AFIDs-OASIS30 dataset

#### Subject demographics and imaging protocol

This subset consists of 30 subjects (age: 58.0 ± 17.9 years; range: 25–91; 17 female) selected from the publicly available Open Access Series of Imaging Studies (OASIS-1) database^14^ and imaged at 3-T. The subjects were cognitively intact (Mini-Mental State Examination = 30), and the MRI scans were specifically chosen to be challenging (areas with more complex anatomy and asymmetries) to ensure the protocol could work over a broad spectrum of structural variability. More details on the selected subjects can be found in a previous study^2^. It is worth noting that this subset of the OASIS-1 dataset includes different subjects from other currently existing subsets in the neuroimaging literature (for instance, the one used in the Mindboggle project^15^).

#### Rater demographics and AFID placements

The AFID protocol was performed a total of three times on this dataset (2,880 fiducials). Nine raters (1 expert and 8 novices) were involved with annotations (3DSlicer 4.8.1). Each scan within this dataset was randomly assigned for annotation by one expert and two novice raters.

### LHSCPD dataset

#### Subject demographics and imaging protocol

The London Health Sciences Center Parkinson’s disease (LHSCPD) dataset currently consists of 40 subjects diagnosed with Parkinson’s Disease (age: 60.2 ± 6.8, range: 38–70; 13 female) with images acquired at University Hospital in London, ON, Canada on a 1.5-T scanner (Signa, General Electric, Milwaukee, Wisconsin, USA). The detailed imaging protocol was described in a previous study^3^. Due to the heterogenous nature of clinical imaging, MRI scans across patients in this dataset are not always consistent in all three dimensions. Ethics approval was received for the anonymized release of patient scans by the Human Subject Research Ethics Board (HSREB) office at Western University (REB# 109045). Patients signed written consent forms for undergoing clinical imaging and open release of this data.

#### Rater demographics and AFID placements

The AFID protocol was performed a total of five times on this dataset (6,400 fiducials). Five raters (2 experts and 3 novices) were involved with annotations (3DSlicer 4.10.0). Each scan within this dataset was annotated by all five raters.

### SNSX dataset

#### Subject demographics and imaging protocol

The Stereotactic Neurosurgery (SNSX) dataset currently consists of 32 healthy participants (age: 46.2 ± 13.5 years; range: 20–70 years; 12 female) with images acquired at the Centre for Functional and Metabolic Mapping (Western University, Canada) on a 7-T head-only scanner (Siemens Magnetom; Siemens Healthineers, Erlangen, Germany). An 8-channel parallel transmit/32-receive channel coil was used. The detailed imaging protocol and pre-processing steps were documented in a previous study^7^. Ethics approval was received for open release of patient scans by the HSREB office at Western University (REB# R-17–156). Patients signed written consent forms for participating and open release of this data.

#### Rater demographics and AFID placements

The AFID protocol was performed a total of three times (3,072 fiducials) on this dataset.

Nine raters (3 experts and 6 novices) were involved with annotations (3DSlicer 4.8.1). Each scan within this dataset was randomly assigned for annotation by one expert rater and two novice raters.

### MNI2009bAsym & Agile12v2016 & MNIColin27 templates

#### Template details and imaging protocol

A group of commonly used public templates were annotated. The *MNI2009bAsym* is a population group template consisting of 152 individuals (age: 18.5–43.5 years) used commonly in the literature^16^. The images were acquired on a Philips 1.5-T Gyroscan (Best, Netherlands) scanner at the Montreal Neurological Institute.

The *Agile12v2016* is an ultra-high field template created locally at our institution. It consists of 12 healthy control subjects (age: 27.6 ± 4.4 years; 6 female). Scans were acquired on a 7-T scanner (Agilent, Santa Clara, California, USA/Siemens, Erlangen, Germany) via a 24-channel transmit-receive head coil array^17^.

The *MNIColin27* is a template created from one subject scanned 27 times on a Phillips 1.5-T MR unit^18^.

#### Rater demographics and AFID placements

The AFID protocol was performed a total of 32 times (1,024 fiducials/template). The same raters who annotated the AFIDs-OASIS30 dataset also annotated all the templates via 3DSlicer 4.8.1. Each template was annotated by eight raters four times.

### BigBrainSym & MNI2009bSym & PD-25 templates

#### Template details and imaging protocol

BigBrain is an ultra-high resolution histological 3D model of the brain created using a large-scale microtome to cut a complete paraffin-embedded brain (65-year-old male) coronally at 20 µm thickness^19^. The BigBrainSym template refers to the BigBrain registered to MNI2009bSym space, defined in previous studies^2,6^. The MNI2009bSym is a symmetric version of the MNI2009bAsym^16^.

The PD-25 template is a multi-contrast MNI template of a PD cohort with 3-T field strength^20^. We used the PD25-T1MPRAGE for the AFID placements.

#### Rater demographics and AFID placements

The AFID protocol was performed a total of two times (64 fiducials/template) by two expert raters via 3DSlicer 4.8.1.

### TemplateFlow templates

#### Template details and imaging protocol

All adult human structural MRI templates that were available on TemplateFlow (see Table 2) at the time of manuscript preparation, which had not been previously annotated, were included (n = 8)^21^.

#### Rater demographics and AFID placements

The AFID protocol was performed a total of four times (128 fiducials/template). Four raters (1 expert and 3 novices) annotated each template once via 3DSlicer 4.8.1.

### AFLE calculation for all datasets and templates

**Fig. 3:**
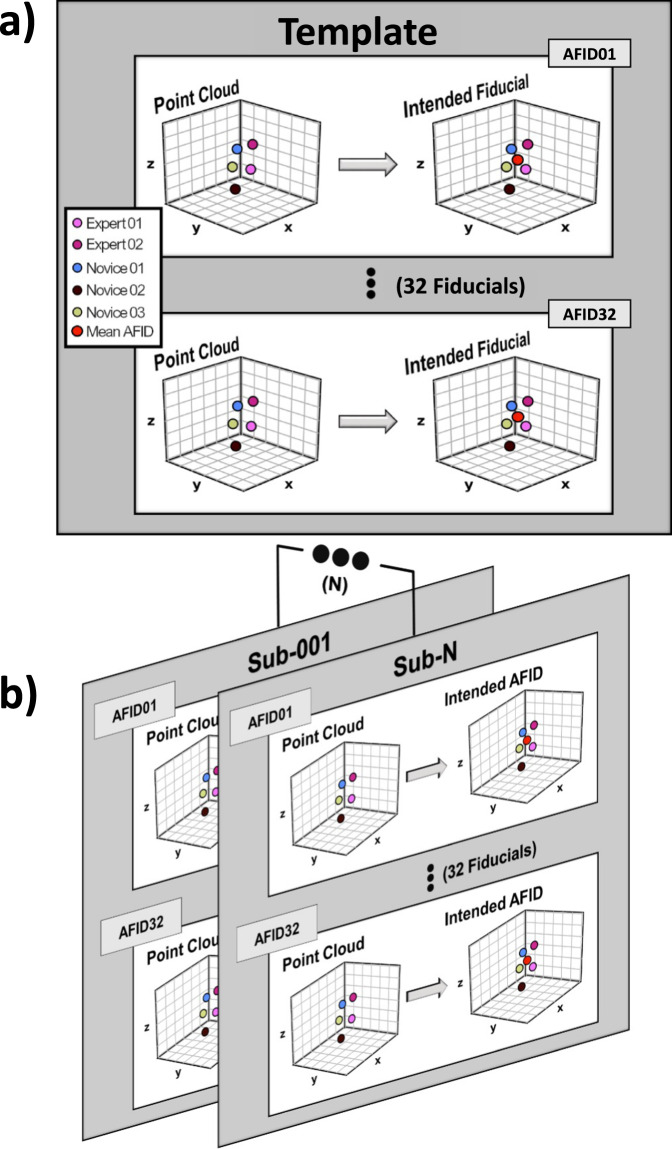
Ground truth anatomical fiducial (AFID) placement on templates and datasets. (**a,b**) show the process of computing the intended AFID placement on a neuroimaging template or dataset, respectively. It is the mean of the rater point cloud at each AFID, referred to as “ground truth” in the text.

All placements for a given scan and fiducial were averaged to achieve the ground truth fiducial placement per participant or template as shown in Fig. 3a. For datasets, ground truth fiducial placements were computed for each subject in a dataset as shown in Fig. 3b.

**Fig. 4:**
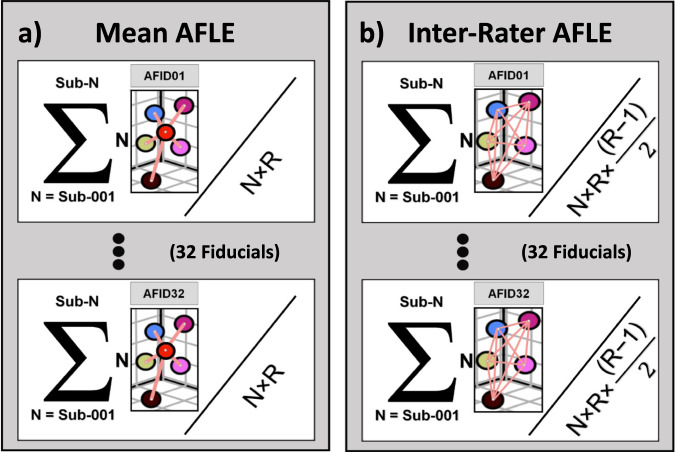
The technical validation computations for our anatomical fiducial (AFID) placements on templates and datasets. (**a,b**) show the equations used to compute mean and inter-rater anatomical localization error, respectively. *N* = *number of subjects in a dataset*. If calculating for a template, *N* would be 1. *R* = *the number of raters per image*. In (**a**) Euclidean distances (shown in pink) represent distance from rater placement to the ground truth (red). The mean AFLE was calculated by dividing the sum of all Euclidean distances across all subjects with the total number of Euclidean distances in the dataset (*N* × *R*) for each AFID. In (**b**) Euclidean distances (shown in pink) represent the pairwise distances between all rater placements on a scan. Inter-rater AFLE was calculated by dividing the sum of the pairwise distances (shown by the sigma notation) by the total number of rater pairwise distances across a dataset per AFID $\left(N\times R\times \frac{\left(R-1\right)}{2}\right)$.

To compute the mean AFLE, Euclidean distances from the ground truth fiducial location to each of the individual rater placements were averaged for each fiducial. The result is termed the subject or template mean AFLE per fiducial. This process was independently repeated for all subjects. All subject mean AFLEs were averaged to achieve a dataset mean AFLE per fiducial as shown in Fig. 4a. Finally, the dataset mean AFLE per fiducial was averaged across all fiducials to produce the global dataset mean AFLE. In a similar fashion, global inter-rater AFLE was computed for one subject across fiducials and then averaged across all subjects to produce a global dataset inter-rater AFLE shown in Fig. 4b.

## Data Records

In total, we release the curated AFID placements and associated imaging of 4 datasets and 14 openly available human brain templates (a total of 19,520 manually placed anatomical landmarks — more than 300 human rater annotation hours).

All datasets have been deposited at DOI-issuing repositories separately (i.e., Zenodo or OpenNeuro)^22^–^25^ and follow the BIDS directory hierarchy (see Table 3 for licensing and metadata). Each dataset’s main directory contains 1) files about licensing and metadata, 2) imaging data directories organized per subject, and 3) a “derivatives” directory which includes AFID coordinates also organized per subject. We release both imaging and AFID annotation data. However, since some of the imaging datasets are protected under Data Usage Agreements (DUAs), the user needs to view and accept terms under DUAs before the imaging data becomes available for download.

**Table 3:** Imaging and anatomical fiducial (AFID) placement metadata in the “afids-data” repository which links the following individual datasets.

Datasets	Repository	DOI	License	
			Imaging	AFID Annotations
AFIDs-HCP30	Zenodo	10.5281/zenodo.8072105	Protected by DUA	Creative Commons (CC BY 4.0)
AFIDs-OASIS30	Zenodo	10.5281/zenodo.7641090	Protected by DUA	
LHSCPD	OpenNeuro	10.18112/openneuro.ds004471.v1.0.0	Creative Commons (CC0)	
SNSX	OpenNeuro	10.18112/openneuro.ds004470.v1.0.0		

For ease of acquiring the data, we merge released datasets and templates in a “super” dataset (https://github.com/afids/afids-data)^26^ which serves as a centralized repository for this data descriptor and can be used to install all of the imaging and AFID annotation data we release.

In brief, the centralized repository (i.e., super dataset) has three main directories, 1) data: raw and curated coordinate files for datasets and templates alongside the MRI scans on which coordinates have been applied in BIDS format, 2) notebooks: code used for data curation and quality control, and 3) other: rater demographics, interactive glass brain showing applied coordinates, and a list of our curated brain landmarks.

Raw rater annotations were released. These files have a “rater” label under the “desc” BIDS entity with session also encoded (if applicable). Additionally, curated mean placements (have a “groundtruth” tag in “desc” BIDS entity) are made available. We make individual rater placements available so users have the opportunity to select the subset of rater placements for their intended application. However, we believe that our “ground truth” placements provide the best estimate as to where the true AFIDs are located.

The AFIDs coordinates are described using the Markups comma-separated values file (i.e., *.fcsv), which is generated after the raters save their placements on 3DSlicer. The *.fcsv file contains coordinate data organized in rows for each of the 32 landmarks of interest, with columns describing the AFID and corresponding x, y, and z coordinates in native subject or template space. As for the imaging data, all dataset images used for annotations were BIDS compatible and made available in a compressed NIfTI-1 format (i.e., *.nii.gz).

We release our anatomical landmark annotations under the Attribution 4.0 International (CC BY 4.0) license, available in *“DERIVATIVE_DATA_USE_AGREEMENT.txt”* file at the level of each dataset. Meanwhile, imaging data are protected by a DUAs which we make available in the *“IMAGING_DATA_USE_AGREEMENT.txt”* file, also at the level of each dataset.

## Technical Validation

As mentioned in the methods, raters typically go through the AFID protocol by referring to the detailed documentation and resources we have made available online (https://afids.github.io/afids-protocol)^12^. To ensure the placements we share are accurate and reproducible amongst expert and novice raters, we computed the AFLE metrics and validated that they are generally within 1–2 mm. Table 4 summarizes the AFLE metrics computed for each of the templates and datasets. Across all AFID protocol performances, the global mean AFLE metric was 0.99 ± 0.32 mm.

**Table 4:** Summary of anatomical fiducial localization errors (AFLE) and Euclidean distances (ED) used for their calculation across released data.

Template or Dataset	EDs utilized for AFLE metrics	AFLE ± Error	
		Mean	Inter-rater
MNI152NLin2009bAsym	Mean: 1,024 and inter-rater: 896	0.99 ± 1.11	1.07 ± 0.46
MNIColin27		1.71 ± 2.78	1.36 ± 0.88
Agile12v2016		1.10 ± 1.59	1.14 ± 0.48
BigBrainSym	Mean: 64 and inter-rater: 32	0.63 ± 0.50	1.25 ± 1.02
MNI152NLin2009bSym		0.55 ± 0.26	1.09 ± 0.52
PD-25		0.42 ± 0.24	0.83 ± 0.47
MNI152Lin	Mean: 128 and inter-rater: 192	1.07 ± 0.45	1.74 ± 0.74
MNI152NLin2009cAsym		1.03 ± 0.40	1.67 ± 0.63
MNI152NLin2009cSym		1.06 ± 0.47	1.67 ± 0.63
MNI152NLin6Asym		1.16 ± 0.51	1.90 ± 0.86
MNI152NLin6Sym		1.08 ± 0.54	1.73 ± 0.84
MNI305		1.14 ± 0.41	1.85 ± 0.52
OASIS30ANTs		0.78 ± 0.33	1.25 ± 0.51
fsaverage		1.00 ± 0.44	1.65 ± 0.73
AFIDs-HCP30	Mean: 2,880 and inter-rater: 2,880	0.66 ± 0.22	1.15 ± 0.70
AFIDs-OASIS30		0.94 ± 0.73	1.58 ± 1.02
LHSCPD	Mean: 6,400 and inter-rater: 12,800	1.57 ± 1.16	2.01 ± 1.49
SNSX	Mean: 3,072 and inter-rater: 3,072	0.96 ± 0.33	1.64 ± 1.37
**Total or Average**	**Mean: 19,520 and inter-rater: 25,952**	**0.99 ± 0.32**	**1.48 ± 0.34**

## Usage Notes

We recommend loading the shared AFID annotation files (*.fcsv) in 3DSlicer alongside their associated images all of which are in BIDS format for ease of navigating. The local neuroimaging datasets we release here (namely, the LHSCPD and SNSX) will be quality controlled and expanded as more participants are recruited. Additionally, new brain landmarks can be added to future versions of the data descriptor once they have met validation standards set by prior related studies^2,3^.

Access to imaging data is granted after users accept the DUAs. Directions on how to gain access have been added for each of the datasets^22^–^25^. For the AFIDs-HCP dataset, users will need to create an account on the HCP website (https://db.humanconnectome.org) and accept the DUA via the portal which will subsequently provide them with an access key to use when cloning our repository. For the AFIDs-OASIS dataset, users will need to accept the DUA on the website (https://www.oasis-brains.org), but no user credentials are required for data access. Permission to reshare imaging data from OASIS and HCP has been acquired. We share imaging data from our locally curated datasets, SNSX^24^ and LHSCPD^25^, under a Creative Commons license so no further user intervention is needed when acquiring those data.

Users can download all the datasets used in this study, after installing DataLad (https://www.datalad.org/#install)^27^ and accepting DUAs, with two lines at the command prompt: “datalad install -r *https://github.com/afids/afids-data.git*” and then *“datalad get -r .”* after migrating to the directory containing installed data. Alternatively, users can download individual datasets or images by calling *“datalad get -r .”* at the directory of interest within the installed dataset. Before images download, the user will be prompted to input relevant information (e.g., user credentials) to ensure the DUAs have been accepted. More granular details on downloading data can be found on our main repository^26^. Although users can acquire the data from the individual dataset directories, we highly recommend using the centralized repository.

## Data Availability

The code used for technical validation as well as prior AFID studies can be found on the GitHub repository page (https://github.com/afids), including the validator tool (https://github.com/afids/afids-validator).
